# Response to “Comment on ‘Predictors of Indoor Radon Concentrations in Pennsylvania, 1989–2013’”

**DOI:** 10.1289/ehp.1510493

**Published:** 2015-11-01

**Authors:** Joan A. Casey, Sara G. Rasmussen, Brian S. Schwartz

**Affiliations:** 1 Robert Wood Johnson Foundation Health & Society Scholars Program, University of California, San Francisco, and University of California, Berkeley, California, USA; 2 Department of Environmental Health Sciences, Johns Hopkins Bloomberg School of Public Health, Baltimore, Maryland, USA; 3 Department of Epidemiology, Johns Hopkins Bloomberg School of Public Health, Baltimore, Maryland, USA; 4 Center for Health Research, Geisinger Health System, Danville, Pennsylvania, USA

We thank Engelder for his interest in our recent publication on indoor radon concentrations in Pennsylvania. We agree that some press coverage about our article was misleading, perhaps due to various interest groups’ concern about radon and shale gas development. Therefore, shortly after publication in *EHP* we used The Conversation, an independent research news website, to clarify our findings (Casey and Schwartz 2015). Our main messages regarding radon have been that geology is the most important contributor, unconventional natural gas development (UNGD) may make a small contribution, and there is a need for continued radon monitoring in states with rapid and continuing UNGD.

Engelder focused his letter on just one of our analyses that related UNGD to building radon levels, the one that categorized counties into five groups. We note that there are two additional analyses that provided parallel evidence, which he did not address.

As Engelder noted, county-category average radon differences were small, but aggregation to the county level may mask larger UNGD-associated differences in smaller geographies or in individual buildings—a question for future research. While we agree that a difference of 0.4 piC/L between two individual measurements may be of little importance, what we observed was a mean difference of approximately 0.4 piC/L among hundreds of thousands of measurements. Aggregated, these small average differences directly translate into more lung cancer.

We also agree with Engelder that Pennsylvania counties contain different geologies, which is why we adjusted for geologic unit in all analyses. Because geology, not UNGD, is the primary driver of indoor radon concentrations, it is not surprising that when we grouped counties the average radon concentrations were not ordered by level of Marcellus activity. Nevertheless, it is not absolute radon levels but rather relative changes over time that might provide insight about impacts from recent UNGD. In our analysis, which accounted for many factors, including geology, we found that after significant UNGD began in 2009, counties with high drilling activity had statistically significantly higher radon levels than counties with no drilling, a divergence from the earlier trends presented in our article. Although an upward trend was evident prior to UNGD, both our county category and overall analysis suggest larger increases after 2009, when UNGD accelerated.

Engelder correctly identified an error in our description of the wells included in our analysis. We stated that we only included horizontally drilled wells, but we included all unconventional wells (both vertical and horizontal) in all analyses. Our data indicate that more than 200 unconventional wells were drilled by 2007, but counts do differ by source.

Engelder suggested that the only pathway for radon from UNGD to enter buildings is through use of natural gas for heating or cooking. With recent studies demonstrating the importance of ambient air and groundwater, we believe these pathways also could play a role. Fugitive emissions can travel long distances, and radioactive ingrowth can lead to increases in radiation from an original emission over many years (Nelson et al. 2015; Tait et al. 2013; Vinciguerra et al. 2015).

Engelder next asserted that statistics from the Pennsylvania Department of Environmental Protection (PADEP) show that summertime radon concentrations “are lower in both basements and first floors,” but our analysis using PADEP data indicated neither basement nor first-floor levels were lower in the summer than in other seasons (Table S2), consistent with our other findings. So, contrary to Engelder’s claim, we did observe elevated first-floor radon concentrations during summer months.

In a second analysis related to UNGD, we also found higher summertime first-floor radon levels in buildings located near a higher density of drilled unconventional wells. There was an attenuated association for basement concentrations, as would be expected if radon associated with UNGD entered on the first floor.

We agree with Engelder that it is possible another factor could explain our findings. However, contrary to Engelder’s suggestion, it is unlikely that soil moisture is one of them, because we adjusted for monthly rainfall in our models. In a third analysis related to UNGD, which Engelder did not address, we observed that buildings with more exposure to producing Marcellus wells had higher concentrations of indoor radon. Since the amount of natural gas produced increased dramatically from 2005 to 2013, we noted that the finding could be explained by another factor that also discernably changed during that time and was associated with UNGD. However, we have no knowledge of other such factors, and certainly none that have changed as dramatically over the past decade as has UNGD in Pennsylvania.

## References

[r1] Casey JA, Schwartz BS. (2015). Small increases in radon track natural gas development with fracking in Pennsylvania. The Conversation, Environment + Energy section (10 April 2015).. https://theconversation.com/small-increases-in-radon-track-natural-gas-development-with-fracking-in-pennsylvania-39991.

[r2] Nelson AW Eitrheim ES Knight AW May D Mehrhoff MA Shannon R 2015 Understanding the radioactive ingrowth and decay of naturally occurring radioactive materials in the environment: an analysis of produced fluids from the Marcellus Shale. Environ Health Perspect 123 7 689 696; 10.1289/ehp.1408855 25831257 PMC4492269

[r3] Tait DR Santos IR Maher DT Cyronak TJ Davis RJ 2013 Enrichment of radon and carbon dioxide in the open atmosphere of an Australian coal seam gas field. Environ Sci Technol 47 7 3099 3104; 10.1021/es304538g 23444905 PMC3621574

[r4] Vinciguerra T Yao S Dadzie J Chittams A Deskins T Ehrman S 2015 Regional air quality impacts of hydraulic fracturing and shale natural gas activity: evidence from ambient VOC observations. Atmos Environ 110 144 150; 10.1016/j.atmosenv.2015.03.056

